# Association of Postpartum Inpatient Consultation Compared With Outpatient Referral With Hepatitis C Virus Treatment Completion

**DOI:** 10.1097/og9.0000000000000112

**Published:** 2025-09-11

**Authors:** Leah M. McCrary, Zoë Leydh, Megan R. Curtis, Jessica Elrod-Gallegos, Patrick Kojima, Lauren Karpman, Jami Cain, Katy Vora, Jahnavi Bongu, Tracey Habrock-Bach, Michael J. Durkin, Cassandra Trammel, Jeannie C. Kelly, Laura R. Marks

**Affiliations:** Division of Infectious Diseases and the Division of Maternal-Fetal Medicine, Washington University in St. Louis School of Medicine, St. Louis, Missouri.

## Abstract

**OBJECTIVE:**

To evaluate the associations of a traditional postpartum outpatient referral compared with an opportunistic inpatient consultation for hepatitis C virus (HCV) treatment initiation on HCV treatment completion.

**METHODS:**

We performed a retrospective evaluation of the HCV perinatal care cascade for women who delivered at Barnes-Jewish Hospital between January 1, 2020, and September 1, 2023. We compared an intervention that provided immediate HCV treatment on discharge after delivery with referral to postpartum outpatient HCV care. Obstetric patients were allocated to either inpatient treatment or outpatient follow-up based on the availability of inpatient consultants. The primary outcome was *treatment completion*, defined as patient-reported completion of direct-acting antivirals (DAAs) or sustained virologic response or negative HCV viral load at 4 weeks or more. The association of the two treatment strategies with treatment completion was assessed using logistic regression.

**RESULTS:**

A total of 149 HCV-affected pregnancies were reviewed. One hundred twenty-five (83.9%) women were referred to HCV treatment during their pregnancies or during their delivery admissions, with 33 (26.4%) in the inpatient referral group and 92 (73.6%) in the outpatient referral group. All individuals in the inpatient referral group had an inpatient consultation; however, a majority of those (55.4%) in the outpatient referral group (92/125; 73.6%) did not present for consultations. Among women who were prescribed DAAs, inpatient referral was associated with higher rates of HCV treatment completion (20/30; 66.7%) compared with standard care (14/41; 34.0%). Inpatient treatment was associated with 4.7 times higher adjusted odds of treatment completion (95% CI, 1.57–14.00).

**CONCLUSION:**

Providing immediate bedside delivery of DAAs at discharge from labor and delivery for women with HCV-affected pregnancies was associated with higher rates of treatment completion than referral for outpatient follow-up. The model of care should be considered for broader implementation.

## Abstract 2

Engaging women in hepatitis C virus care by providing direct-acting antivirals on discharge from labor and delivery represents an important opportunity to increase engagement in hepatitis C virus treatment.

Globally, women of childbearing age make up a quarter of the world's population and account for more than one-fifth of chronic hepatitis C virus (HCV) infections.^1^ In the United States, the prevalence of HCV infection among women presenting for delivery increased 10-fold between 2000 and 2019, to 0.49%.^1^ Among these delivering patients, 8% of neonates acquired perinatal HCV infection.^2^ Hepatitis C virus infection has been associated with adverse pregnancy and neonatal outcomes such as intrahepatic cholestasis of pregnancy, preterm labor, and hemorrhage.^3,4^

Direct-acting antivirals (DAAs) are well-tolerated and highly effective, achieving sustained virologic response (SVR) and curing more than 95% of patients.^5^ However, despite the recent implementation of routine HCV screening in pregnancy,^6^ pregnant and postpartum women represent a special population in which treatment access remains limited despite increasing prevalence.^7^ Among women with pregnancies complicated by HCV infection and opioid use disorder (OUD), fewer than 6% were prescribed DAAs within 6 months of delivery.^8^

The postpartum period can be a challenging time, with low maternal health care appointment attendance.^9,10^ Hospitalization has been shown to be an important opportunity to initiate HCV treatment among nonpregnant individuals.^11^ Our objective was to compare HCV treatment completion rates between a program for providing postpartum patients with DAA prescription before the time of hospital discharge ( Meds to Beds program) and standard postpartum outpatient referral (usual care).

## METHODS

In this single-center, retrospective cohort study, we analyzed electronic health record (EHR) data for all patients who delivered at Barnes-Jewish Hospital—an urban Midwestern tertiary care center that offers level IV perinatal care—from January 1, 2020, to September 30, 2023, tested positive for HCV infection, and were HCV-treatment naive. *Hepatitis C virus infection* was defined as a positive HCV RNA test result during pregnancy or on admission to the labor and delivery unit. We included women with no history of hepatitis B virus or human immunodeficiency virus (HIV) co-infection. Beginning in 2020, our institution implemented shared decision making to offer DAAs to women who may choose to breastfeed. Women were counseled regarding risks, benefits, and lack of safety data of DAAs with breastfeeding and were supported in breastfeeding while on DAAs if they chose to do so.

Hepatitis C virus testing during pregnancy became a standard of care at Barnes-Jewish Hospital after publication of the American Association for the Study of Liver Diseases and Centers for Disease Control and Prevention recommendations for prenatal screening in early 2020.^12^ All obstetric residents received education on HCV infection, including treatment with DAAs, as part of their core curriculum and integrated prompts for additional workup for guideline-recommended HCV pretreatment laboratory studies^13^ into their standardized templates. Because all patients were treated with pan-genotypic agents, viral genotyping was not required to guide treatment. Due to fluctuating resource availability and practical constraints, participants were nonrandomly allocated to one of the two care pathways based on the availability of the infectious disease consultant. Inpatient treatment (Meds to Beds) was offered on weekdays, when an infectious disease consultant with HCV expertise was available. When inpatient consultation was not available, obstetric teams were instructed to place an outpatient referral order and an infectious disease case manager assisted with scheduling the patient and arranging for follow-up.

In the Meds to Beds program, an infectious diseases physician would see a patient during their admission for labor and delivery to counsel regarding the natural history of HCV infection and the effectiveness of treatment and to ensure that guideline-directed pretreatment laboratories were completed. Once the necessary laboratory results were available, DAAs were prescribed to be started at the time of discharge from the hospital. Specialty infectious diseases pharmacists assisted with prior authorizations, reviewed formulary lists, and evaluated for drug interactions. Direct-acting antivirals were brought to the bedside by the subspecialty outpatient pharmacy at the time of hospital discharge. For individuals with insurance requirements necessitating use of another pharmacy, the subspecialty pharmacist would assist with completing the prior authorization before transferring the prescription to the insurance-selected pharmacy. Patients were provided with the external pharmacy information and assistance with arranging for medications to be delivered to their homes so they could start HCV treatment after discharge. All treated individuals were offered follow-up visits.

We collected laboratory and medication data, problem lists, social history data fields, demographics, and visit data through the EHR. Detailed demographics, obstetric outcomes, HCV treatment, and delivery outcomes were abstracted from the EHR by trained research staff. Race was included in this analysis as a covariate because of its established association with decreased access to HCV treatment.^14^ We assessed breastfeeding at time of discharge and attendance at any postpartum visit for HCV treatment. Obstetric outcomes included intrahepatic cholestasis of pregnancy, preterm delivery, and intrauterine fetal death. Neonatal charts were reviewed for adverse outcomes including neonatal death and vertical HCV transmission. Neonatal charts of mothers who were breastfeeding on DAAs were reviewed for any adverse neonatal outcomes, including any transaminitis or neonatal death.

Our primary outcome was HCV treatment completion. We used descriptive statistics to characterize the HCV care pathway, including time (days) from delivery to the first recorded HCV treatment prescription. We defined the HCV care pathway as follows: 1) women with *HCV-affected pregnancies*, defined as having a positive HCV RNA test result at any time during pregnancy or delivery; 2) referral for HCV treatment as evidenced by referral to an HCV treatment physician, scheduling an appointment with an HCV treatment physician, or a consult order for an inpatient consult with an HCV treatment physician; 3) HCV-related consult or presentation to an outpatient clinic or telemedicine appointment to discuss HCV treatment; 4) HCV DAA ordered, as evidenced by a prescription order in the EHR; and 5) successful HCV treatment completion, as evidenced by either SVR at 4 weeks or more (SVR4) or patient report of DAA completion in individuals who were unable to complete laboratory testing due to poor venous access.

An a priori sample size calculation was not performed because sample sizes were fixed. Demographic and outcomes data were compared between groups using the Mann-Whitney *U*, χ^2^, or Fisher exact test for dichotomous variables, as appropriate. Univariable models evaluated the following covariates that clinically we expected to affect HCV treatment rates: race; substance use (categorical variable encompassing problem list and social history data); *engagement in prenatal care* (defined as attending more than two office visits with an obstetrics office during the pregnancy); *rural or urban status* as defined by rural–urban commuting area codes; and insurance status at delivery (Medicaid, non-Medicaid, self-pay).

We calculated absolute risk difference by subtracting the proportion of women who completed HCV treatment in the usual care group from the proportion in the Meds to Beds group. The number needed to treat was derived as the inverse of the absolute risk difference. We performed a subgroup analysis evaluating completion rates among women with limited engagement in prenatal care. An association between treatment strategy and HCV treatment completion was evaluated using logistic regression, adjusting multivariable models for variables identified a priori for face validity such as categorical age (25–29 years, 30–34 years, 35–39 years, and 40–46 years), Medicaid status, history of illicit substance use, and engagement in prenatal care.

This study was approved by the Washington University in St. Louis IRB. Stata was used for all analyses. *P*<.05 was considered statistically significant.

## RESULTS

Demographics and characteristics of the 149 pregnancies among treatment-naive women with HCV infection included in the study period are shown in Table 1. A total of 94.6% of women reported substance use during pregnancy, with 74.5% having a diagnosis of OUD complicating pregnancy. Medications for OUD were prescribed to 42.3% of women with OUD. Limited prenatal care was common, with 53.7% of women attending two or fewer outpatient prenatal visits. Hepatitis C virus–related complications noted in this cohort included intrahepatic cholestasis of pregnancy (2.7%) and preterm delivery (34.2%). Of exposed neonates, 28.5% were tested. Perinatal transmission of HCV infection occurred in 4 of 41 neonates (9.8%) with available HCV testing data. One of our cases involved a dichorionic–diamniotic twin pregnancy in which perinatal transmission was discordant: twin B acquired HCV infection, but twin A did not. Additionally, 87% of women in our cohort of HCV-affected pregnancies previously had at least one pregnancy affected by HCV without treatment in the interpregnancy interval.

**Table 1.** Maternal Demographic Characteristics and Neonatal Outcomes for All Patients With Hepatitis C Virus–Affected Pregnancies (N=149)

Characteristics and Outcomes	Value
Age (y)	34 (27, 41)
Race	
African American	42 (28.3)
White	106 (71.1)
None of the above	1 (0.7)
Hispanic ethnicity	3 (2.0)
Rural residence	29 (19.5)
Insurance (primary payer)	
Medicaid	121 (81.2)
Comorbidities and pregnancy complications	
Substance use during pregnancy	141 (94.6)
OUD	111 (74.5)
On MOUD (buprenorphine or methadone)*	63 (42.3)
Cirrhosis	1 (0.7)
Limited prenatal care^†^	80 (53.7)
Intrahepatic cholestasis of pregnancy	4 (2.7)
Preterm delivery	51 (34.2)
Intrauterine fetal death	4 (2.7)
Neonatal and infant outcomes	
Neonatal death	4 (2.7)
Infant HCV testing performed^‡^	41/144 (28.5)
HCV perinatal transmission^§^	4 (9.8)

OUD, opioid use disorder; MOUD, medications for opioid use disorder; HCV, hepatitis C virus.

Data are median (interquartile range) or n (%).

* Proportion of women on MOUD is calculated as a subset of individuals with OUD.

† Two or fewer outpatient prenatal visits.

‡ Reported for liveborn infants who survived the neonatal period and excludes intrauterine fetal death and neonatal death. One pregnancy was a twin pregnancy.

§ Proportion of tested liveborn neonates.

**Fig. 1.**
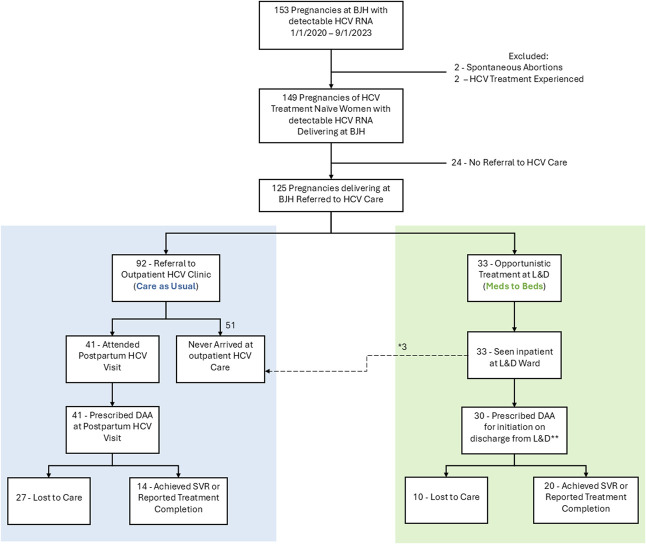
Flow diagram. *Three patients were unable to receive direct-acting antiviral (DAA) prescriptions at the time of discharge from labor and delivery due to either laboratory results not yet available or self-pay status requiring patient-assistance paperwork. **Twenty-eight patients received medications delivered to labor and delivery at the time of discharge, and two had prescriptions sent to a mail-order pharmacy and shipped to their homes immediately after discharge. BJH, Barnes-Jewish Hospital; HCV, hepatitis C virus; SVR, sustained virologic response.

Women were referred to HCV care in 83.9% of affected pregnancies (Fig. 1). No cases of spontaneous clearance were observed among women who did not receive HCV treatment, though subsequent HCV RNA testing was rare. Table 2 lists the demographic and clinical characteristics of women referred to each HCV care pathway (usual care vs Meds to Beds). There was no significant difference in race, ethnicity, rural residence, insurance, substance use during pregnancy, OUD, receipt medications for OUD, or prenatal care engagement between women who were referred to HCV treatment through usual care and those in the Meds to Beds group.

*McCrary. Inpatient Consultation for Postpartum HCV Care. O&G Open 2025*.

**Table 2.** Maternal Demographic Characteristics and Outcomes of Patients Referred to Hepatitis C Virus Care

Characteristics and Outcomes	Usual Care Pathway (n=92)*	Meds to Beds Pathway (n=33)^†^	*P*
Age (y)	33 (26, 40)	33 (29, 37)	.55
African American race	27 (29.4)	6 (18.2)	.21
Hispanic ethnicity	2 (2.2)	1 (3.0)	.78
Rural residence	20 (21.7)	7 (21.2)	.95
Insurance (primary payer)			
Medicaid	76 (82.6)	26 (78.8)	.63
Comorbidities and pregnancy complications			
Substance use during pregnancy	85 (92.4)	32 (97.0)	.36
OUD	65 (70.7)	25 (75.8)	.58
On MOUD (buprenorphine or methadone)^‡^	38 (58.5)	13 (52.0)	.58
Cirrhosis	1 (1.1)	0 (0.0)	.55
Limited prenatal care^§^	44 (47.8)	17 (51.5)	.72
HCV care cascade outcomes			
Attended an HCV-focused visit (inpatient consultation or outpatient visit)	41 (44.6)	33 (100)	<.01
Prescribed DAA	41 (44.6)	30 (90.9)	<.01
Reported completing DAAs or achieved SVR	14 (15.22)	20 (60.6)	<.01

OUD, opioid use disorder; MOUD, medications for opioid use disorder; HCV, hepatitis C virus; DAA, direct-acting antiviral; SVR, sustained virologic response.

Data are median (interquartile range) or n (%) unless otherwise specified.

* Referred to postdischarge HCV treatment.

† Referred to inpatient consultation with an infectious diseases specialist for opportunistic treatment of HCV at labor and delivery.

‡ Calculated as a subset of individuals with OUD.

§ Two or fewer outpatient prenatal visits.

**Fig. 2.**
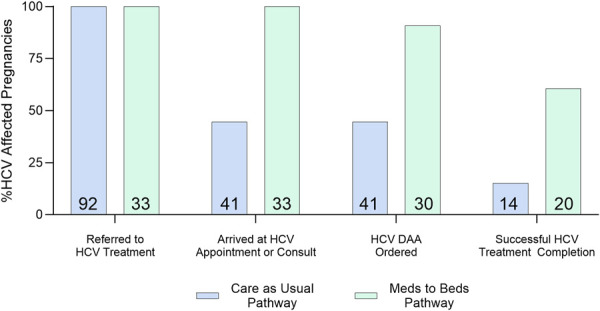
Perinatal hepatitis C virus (HCV) care cascade of women referred to HCV care by treatment strategy. Percentages calculated from all individuals referred to HCV treatment for each pathway (n=92 for usual care pathway, n=33 for Meds to Beds pathway). *Numbers at base of bars* indicate total number in each stage of the care cascade. DAA, direct-acting antiviral.

Figure 2 displays the HCV care cascade by HCV treatment strategy. Of the 125 pregnant patients who were referred for HCV care, 92 (73.6%) were referred to outpatient HCV treatment (usual care).

*McCrary. Inpatient Consultation for Postpartum HCV Care. O&G Open 2025*.

A majority (55.4%) of the women in the usual care pathway were lost to follow-up between discharge from labor and delivery and attending an HCV-focused clinic visit. All patients who attended an HCV-focused outpatient appointment received a DAA prescription. The median time between delivery and HCV prescription was 57 days in the usual care group and 2 days in the Meds to Beds group (Table 3).

**Table 3.** Demographic Characteristics of Patients Who Received an Hepatitis C Virus Consultation and Received a Prescription for a Direct-Acting Antiviral for Hepatitis C Virus Treatment, by Treatment Group

Characteristic	Usual Care Pathway (n=41)*	Meds to Beds Pathway (n=30)^†^	*P*
Age (y)	33 (26, 40)	33 (29, 37)	.75
African American race	8 (19.5)	6 (20.0)	.96
Hispanic ethnicity	0 (0.0)	1 (3.3)	.24
Rural residence	10 (24.4)	6 (20.0)	.66
Insurance (primary payer)			
Medicaid	31 (75.6)	24 (80.0)	.66
Comorbidities and pregnancy complications			
Substance use during pregnancy	37 (90.3)	29 (96.7)	.30
OUD	25 (61.0)	23 (76.7)	.16
On MOUD (buprenorphine or methadone)^‡^	18 (72.0)	12 (52.2)	.16
Cirrhosis	0 (0.0)	0 (0.0)	—
Limited prenatal care^§^	15 (36.6)	14 (46.7)	.40
Intrahepatic cholestasis of pregnancy	3 (7.3)	0 (0.0)	.13
Term delivery	29 (70.7)	19 (63.3)	.51
Breastfeeding	13 (31.7)	10 (33.3)	.89
HCV treatment characteristics and outcomes			
Attended a follow-up HCV focused encounter	5 (12.2)	5 (16.7)	.60
Days from delivery to HCV prescription	30 (17, 43)	1 (0, 2)	<.01
Reported completing DAAs or achieved SVR	14 (34.2)	20 (66.7)	<.01

OUD, opioid use disorder; MOUD, medications for opioid use disorder; HCV, hepatitis C virus; DAA, direct-acting antiviral; SVR, sustained virologic response.

Data are median (interquartile range) or n (%) unless otherwise specified.

* Referred to postdischarge HCV treatment.

† Referred to inpatient consultation with an infectious diseases specialist for opportunistic treatment of HCV at labor and delivery.

‡ Calculated as a subset of individuals with OUD.

§ Two or fewer outpatient prenatal visits.

In the inpatient treatment pathway (Meds to Beds), all 33 women with HCV infection were evaluated by an infectious disease specialist before discharge from their delivery hospitalization. Of these, 30 were discharged with a prescription for HCV treatment with a DAA, and 28 (84.9%) were discharged from labor and delivery with DAAs in hand. Two individuals had insurance that required the use of another specialty pharmacy that could not deliver medication to the bedside but allowed for medication delivery to be arranged to their homes. Women in the Meds to Beds pathway were sent to a variety of dispositions, including jail, substance use disorder treatment centers, or their own homes. Three individuals were not immediately started on DAAs due to short hospital admissions during which laboratory testing had been started but results had not yet been returned or because the patients needed to complete a patient-assistance program application for DAAs due to self-pay status. All were offered a postdischarge office or telemedicine-based visit with an infectious diseases specialist, with a documented plan to offer HCV treatment once laboratory results had returned or when information needed for DAAs was available. Unfortunately, none of those three individuals attended that visit.

There were no significant differences among baseline characteristics between women who received a DAA prescription through the Meds to Beds or usual care pathways (Table 2). The Meds to Beds pathway was associated with a statistically significant increase in HCV treatment completion (20/30; 66.67%) compared with usual care (14/41; 34%) (*P*<.05), with a risk difference of 32.5% (95% CI, 10.3–54.8; number needed to treat=3). Moreover, among a subgroup of women who had not engaged in prenatal care, the majority in the Meds to Beds group (9/14; 64%) completed treatment compared with one-fifth (3/15; 20%) of women referred to outpatient follow-up (*P*<.05).

**Fig. 3.**
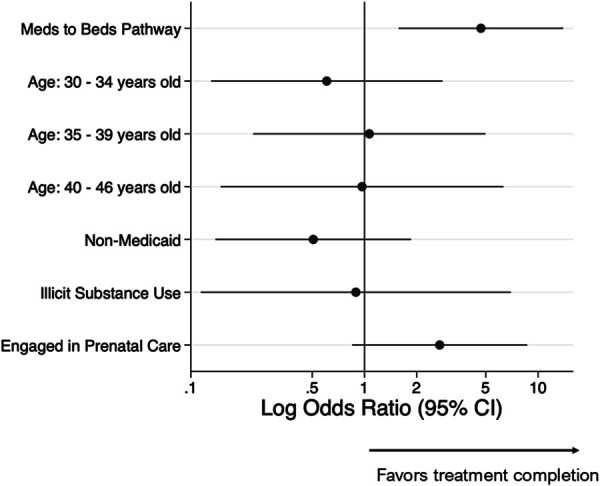
Log-adjusted odds ratio of treatment outcome among women who received a prescription for a direct-acting antiviral for hepatitis C virus treatment.

Figure 3 displays the adjusted odds ratios of HCV treatment completion among women who received a prescription for a DAA. The Meds to Beds pathway was associated with 4.7 (95% CI, 1.57–14.00) times the odds of HCV treatment completion when compared with usual care (Fig. 3) when adjusted for age, Medicaid status, history of illicit drug use, and engagement in prenatal care.

*McCrary. Inpatient Consultation for Postpartum HCV Care. O&G Open 2025*.

Rates of follow-up visits after initiating DAA were low across both groups, with only 5 of the 30 women (16.7%) in the Meds to Beds pathway attending a follow-up appointment and 5 of the 41 (12.2%) in the usual care group.

Because relatively few women attended follow-up HCV visits after DAA initiation, SVR4 laboratory results were abstracted from subsequent visits for which they were available in the EHR. Of the women who did not attend follow-up, 29.5% had SVR laboratory results available in the medical record. These HCV RNA laboratory tests were obtained in a variety of settings, such as initial obstetric laboratory tests during a subsequent pregnancy, intake to the department of corrections, laboratory results at plasma donation centers, or laboratory results during subsequent hospital encounters (emergency department visits or inpatient admissions) for non–HCV-related issues.

Many patients were unable to breastfeed due to current active substance use, but 23 patients chose to breastfeed or pump while on DAAs, including two patients on sofosbuvir and velpatasvir and 21 patients on glecaprevir and pibrentasvir. There were no documented adverse outcomes associated with breastfeeding on DAAs in our cohort. Specifically, there were no cases of transaminitis among infants born to mothers who breastfed while on DAAs. In contrast, the background rate of infant death among those born to mothers in this cohort who were formula feeding or not on HCV treatment due to loss to follow-up was 2.6%.

## DISCUSSION

Our program demonstrates that admission for labor and delivery can be a reachable moment for immediate HCV treatment initiation in the perinatal HCV care cascade. Among individuals seen as inpatient consultations through the Meds to Beds pathway, 90% could be prescribed DAAs at discharge. Immediate treatment with medications delivered to the bedside on discharge from labor and delivery was associated with a higher rate of treatment completion (67%) when compared with individuals referred for outpatient follow-up (34%). On the other hand, more than half of the individuals who were referred to HCV treatment through the usual care pathway were lost to care before initiating DAAs.

Our Meds to Beds program provides a pragmatic model for opportunistic HCV treatment of obstetric patients after delivery. Opportunistic treatment programs focused on labor and delivery hospital admissions can engage women who may have never participated in prenatal care and who might be unlikely to attend postpartum outpatient visits. Using inpatient hospitalization as a touchpoint for HCV care is an emerging opportunity for HCV screening, diagnosis, and linkage-to-care with DAA started inpatient or on discharge. This approach is supported by a randomized trial, the OPPORTUNI-C study,^11^ as well as implementation studies in the United States^15,16^ and Canada.^17^ The care model described in our study of DAA receipt on discharge captures a unique population with outcomes comparable with those in the OPPORTUNI-C trial.

This work was completed in a large academic health care system with a subspecialty infectious diseases pharmacy accustomed to assisting with prior authorizations for DAAs. However, given the widespread removal of prior authorization requirements for DAAs among individuals with Medicaid insurance in more than 50% of U.S. states,^18^ many aspects of this intervention could be replicated by other hospitals. Established bedside medication delivery services are common at large hospitals and are provided in partnership with their outpatient pharmacies, which can access formulary information and coordinate prior authorizations. Hepatitis C virus prescription on discharge can build on these foundations and be widely implemented across U.S. health care systems that already possess established Meds to Beds programs.

Notably, 87% women in our cohort had prior pregnancies that were affected by HCV. Although all were HCV-treatment naive, in several instances, they had been seen previously by HCV clinicians but were recommended to defer postpartum HCV treatment until cessation of breastfeeding. This resulted in limited windows for HCV treatment with DAAs. The American Academy of Pediatrics and the WHO recommend exclusive breastfeeding for the first 6 months of life, followed by continued breastfeeding with complementary foods for at least 2 years and beyond as mutually desired.^19,20^ The historical guidance of deferring DAA treatment until after the end of lactation may play a role in the low postpartum treatment rates seen in national cohorts; retaining women in care until weaning can present challenges for both women and HCV clinics, because lactation may be of unpredictable duration. Ongoing implementation of immediate postpartum treatment regardless of feeding choice has the potential to reduce lost-to-care rates, increasing completion of HCV treatment and, thereby, reducing the risk of perinatal HCV exposure in subsequent pregnancies.

Although our study assesses a novel and promising approach to peripartum HCV linkage to care and treatment, we acknowledge several limitations. First, this was a retrospective cohort, with a significant amount of missing data due to patient loss to care. Second, the care cascade leveraged committed obstetric clinicians who were knowledgeable about initial workup for HCV treatment and championed access to care for their patients. It required same-day consultations to patients who had short admissions (median 3 days) and relied on a pharmacy with personnel and resources to deliver medications to the bedside or the home. The program's success was dependent on the widespread availability of DAAs for patients with Missouri Medicaid insurance without prior authorization or sobriety restrictions, offering immediate day of discharge access for patients who received a DAA prescription. This access is not available in all states.^18^ Lastly, obtaining HCV RNA SVR laboratory results was likely enhanced by ongoing testing initiatives that prompt clinicians to order routine HCV RNA testing on patients with a history of substance use disorders.

Engaging women in HCV care by providing DAAs on discharge from labor and delivery was associated with higher rates of HCV treatment completion when compared with standard outpatient follow-up. This represents an important opportunity in peripartum care to increase engagement in HCV treatment among reproductive aged women and break the cycle of perinatal transmission to improve both maternal and infant health outcomes.
